# Potts’ Disease of the Spine

**Published:** 1876-04

**Authors:** 


### Our Exchanges.

---

Pott's Disease of the Spine—Treatment by Means of the Plaster-of-Paris Jacket in Bellevue Hospital.—This method of treatment just now is very promising. It is simple, is within the reach of every surgeon, and thus far the results attained have been eminently satisfactory. The jacket is applied in the following manner: Have the patient put on a light wrapper, and then by means of a pulley attached to straps which pass through the axillæ and secured to a cross-bar over the head, suspend him completely, or as near so as possible. In this way the weight of the body is made to act as a counter-extending force, and removes all pressure from the diseased surfaces of the bones. Now, while suspended, draw the wrapper down smoothly over the trunk and hips, and then with a roller bandage filled with plaster begin two or three inches below the crests of the illii, sufficient to secure a firm hold upon the pelvis; first making two or three extra turns of the roller at this point, and then embrace the body by successive turns until the entire trunk is encased to the armpits. If the disease is high up in the dorsal region, the bandage can be carried over each shoulder like a pair of suspenders. It is proper to place a roll of cotton upon each side of the spinal column, opposite the seat of disease; and the bandages may be reinforced by strips of tin made rough like a grater to prevent them from sliding, if the size of the patient or other conditions seem to demand it. Two or three rollers are usually sufficient to make a substantial jacket, particularly if they are supported by the tin strips. There is one point with reference to padding the trunk with cotton or other yielding material before applying the roller, and that is, do not pad any more than is absolutely necessary, for in so doing the irregularities of the surface are almost entirely obliterated, which is just the thing to be avoided. It is the gentle molding of this jacket to the depressions and elevations upon the surface of the body that enables it to accomplish its work and be worn without complaint; whereas, if the trunk is padded with cotton to any great extent, the jacket cannot get sufficient hold to keep the pressure off the diseased surfaces, consequently is thrown aside as inefficient. The straightening out of the patient, so to speak, can be done in any mânner the ingenuity of the surgeon may devise. If the patient is a child, for instance, this can be easily accomplished by placing him across the lap of an assistant, who shall separate his thighs until all the extension is made which is necessary. Now, it will be suggested, can respiration be carried on properly while the trunk is encased in such a fixed jacket? Experience so far has proven that nature will take care of that. Indeed, it seems as though a very great benefit is to be derived by keeping the ribs quiet, for they are thus prevented from producing constant rubbing *at or near the seat of the disease in the spinal column.* Again, suppose abscesses are present, how are they to be managed? Simply make a window in the splint, and then the discharge is placed at the command of the surgeon as well as though no jacket were present. Suppose the patient complains of the jacket, that it hurts him, that it does not give him ease from pain, etc.? Remove it at once. Find out where the difficulty is, and remedy it while applying another; for it may be said with a very great amount of certainty that the fault is with the surgeon and not with the plan of treatment. Now, all these points were illustrated in a bright little girl before us, aged twelve years, who had been wearing one of the best constructed braces that American or foreign ingenuity has produced, but which failed to afford the relief desired. Since the plaster-of-paris jacket has been applied she can ride in the cars and walk with perfect ease and comfort, and has gained several pounds in weight.—*New York Medical Record.*

